# The efficacy of anti-PD-1/PD-L1 therapy and its comparison with EGFR-TKIs for advanced non-small-cell lung cancer

**DOI:** 10.18632/oncotarget.18406

**Published:** 2017-06-08

**Authors:** Zhixin Sheng, Xu Zhu, Yanhua Sun, Yanxia Zhang

**Affiliations:** ^1^ Department of Hematology, Weifang People's Hospital, Weifang, China; ^2^ Department of Oncology, Linyi People's Hospital, Linyi, China

**Keywords:** anti-PD-1/PD-L1 therapy, atezolizumab, pembrolizumab, nivolumab, NSCLC

## Abstract

**Purpose:**

To better understand the efficacy and safety of anti-PD-1/PD-L1 therapy (atezolizumab, pembrolizumab, nivolumab) in patients with previously treated advanced non-small-cell lung cancer (NSCLC).

**Methods:**

The Cochrane Controlled Trial Register, Embase, Medline, and the Science Citation Index were searched for prospective published reports of atezolizumab, pembrolizumab, nivolumab in previously treated patients with advanced NSCLC.

**Results:**

Finally, we identified 14 prospective published reports including four trials of atezolizumab covering 542 subjects, three trials of pembrolizumab covering 1566 subjects, seven trials of nivolumab covering 1678 subjects. When compared to docetaxel, anti-PD-1/PD-L1 therapy could significantly improve overall survival (hazard ratio [HR] 0.67, P<0.001) and progression-free survival (HR 0.83, P=0.002) for previously treated patients with advanced NSCLC. Anti-PD-1/PD-L1 therapy produced an overall response rate of 19% in the 2374 evaluable patients. When using docetaxel as the common comparator, indirect comparison of anti-PD-1/PD-L1 therapy versus EGFR-TKIs showed progression-free survival benefit (HR 0.62, P<0.001) and overall survival benefit (HR 0.60, P<0.001) for those patients with EGFR wild-type. Meanwhile, for those EGFR mutant patients, indirect comparison indicated that anti-PD-1/PD-L1 therapy was inferior to EGFR-TKIs therapy in terms of progression-free survival (HR 3.20, P<0.001), but no survival difference (HR 1.30, P=0.18).

**Conclusion:**

Anti-PD-1/PD-L1 therapy could produce progression-free survival and overall survival improvement over docetaxel for patients with previously treated NSCLC. For EGFR wild-type patients, anti-PD-1/PD-L1 therapy seemed to prolong progression-free survival and overall survival when compared to EGFR-TKIs. Meanwhile, for these EGFR mutant patients, anti-PD-1/PD-L1 therapy was inferior to EGFR-TKIs therapy in terms of progression-free survival.

## INTRODUCTION

The interaction of programmed death 1 (PD-1) with the non-small-cell lung cancer (NSCLC) expressed ligands programmed death-ligand 1 (PD-L1) and PD-L2 could downregulate T cell activity and promote tumor immune escape [1–4]. Recently, anti-PD-1/PD-L1 therapy (atezolizumab, pembrolizumab, nivolumab) which could disrupt PD-1/PD-L1-mediated signaling and restore antitumor immunity had been reported to be a good treatment option for advanced NSCLC [5–18]. However, whether anti-PD-1/PD-L1 therapy could provide progression free survival (PFS) improvement still remained undefined for previously treated patients with advanced NSCLC. PFS improvement was only shown in two of the four trials [5–8]. With these results variable, the meta-analysis tried to evaluate the activity and safety of anti-PD-1/PD-L1 therapy in previously treated advanced NSCLC. The primary endpoints were PFS, overall survival (OS), overall response rate (ORR) derived from anti-PD-1/PD-L1 therapy.

Epidermal growth factor receptor tyrosine kinase inhibitors (EGFR-TKIs) such as gefitinib and erlotinib have been used as suggested for heavily pretreated molecularly selected patients with NSCLC [19–22]. Both anti-PD-1/PD-L1 therapy and EGFR-TKIs are considered as vital breakthroughs in the management of advanced NSCLC and are credited for changing this once dismal history of previously treated advanced NSCLC. However, direct head-to-head comparison between EGFR-TKIs and anti-PD-1/PD-L1 therapy is lacking. When a direct comparison is not available, another way to assess the relative activity of competing regimens is to undertake an indirect comparison. Thus, we applied an adjusted indirect comparison analysis to evaluate the relative activity of anti-PD-1/PD-L1 therapy versus EGFR-TKIs for previously treated patients with advanced NSCLC using common comparator.

## RESULTS

### Characteristics of the published reports

**Figure 1:**
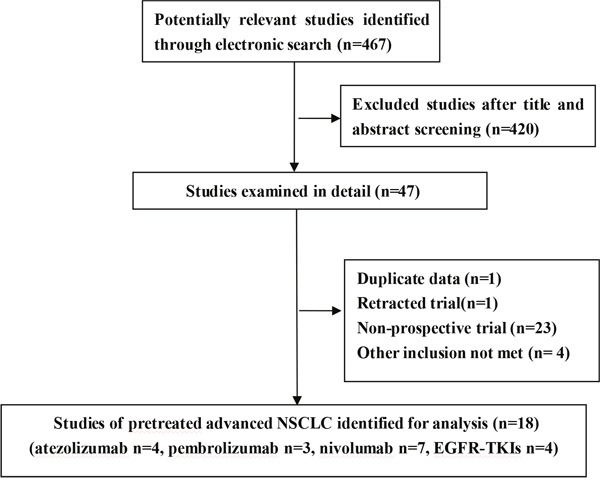
Study flow diagram

Finally, we identified 14 prospective published reports including four trials of atezolizumab covering 542 subjects, three trials of pembrolizumab covering 1566 subjects, seven trials of nivolumab covering 1678 subjects. And, four trials of EGFR-TKIs covering 2475 subjects were also included for indirect comparison [5–22]. All RCTs reported intention-to-treat analyses, description of dropouts, and generation of allocation sequence, however none of them was blind [5–8, 19–22]. Study flow diagram was indicated in Figure 1. Table 1 summarized the characteristics of 18 identified clinical reports.

**Table 1:** Main characteristics of the studies included in the meta-analysis

Study name (year)	N	n		Therapy regimen	Age median	Smoker/non-smoker	Trial type
		EGFR^−^	EGFR^+^				
**Anti-PD-1/PD-L1 therapy Trials**							
Fehrenbacher 2016 [5]	287	-	-	Ate 1200mg, q3w	62	117/27	RCT
				Doc 75mg/m2, q3w	62	114/29	
Herbst 2016 [6]	1033	875	86	Pem 2mg/kg or 10mg/kg, q3w	63	564/123	RCT
				Doc 75mg/m2, q3w	62	269/67	
Borghaei 2015 [7]	582	340	82	Niv 3 mg/kg, q2w	61	231/58	RCT
				Doc 75mg/m2, q3w	64	227/60	
Brahmer 2015 [8]	272	-	-	Niv 3 mg/kg, q2w	62	121/10	RCT
				Doc 75mg/m2, q3w	64	129/7	
Herbst 2014 [9]	53	-	-	Ate 0.3, 1, 3, 10 and 20mg/kg, q3w;10, 15 or 20 mg/kg, q3w	60	68/17	Single-arm
Spigel 2015 [10]	114	-	-	Ate 1200mg, q3w	-	-	Single-arm
Horn 2015 [11]	88	-	-	Ate 20 mg/kg q3w	-	-	Single-arm
Garon 2015 [12]	495	-	-	Pem 2mg/kg or 10mg/kg, q3w;10mg/kg, q2w	64	369/126	Single-arm
Gandhi 2014 [13]	38	-	-	Pem 10mg/kg, q3w	-	-	Single-arm
Gettinger 2015 [14]	129	-	-	Niv 1-, 3-, or 10-mg/kg, q2w	65	-	Single-arm
Rizvi 2015 [15]	117	-	-	Niv 3mg/k, q2w	65	108/9	Single-arm
Brahmer 2012 [16]	49	-	-	Niv 0.3, 1, 3, and 10 mg/kg, q2w	65	-	Single-arm
Topalian 2012 [17]	122	-	-	Niv 1, 3, and 10 mg/kg, q2w	65	-	Single-arm
Gettinger 2014 [18]	20	-	-	Niv 3mg/k, q2w	-	-	Single-arm
**EGFR-TKIs Trials**							
Maruyama 2008 [19]	489	26	31	Gef 250 mg/d	-	174/71	RCT
				Docl 60mg/m2, q3w	-	157/87	
Douillard 2010 [20]	1466	253	44	Gef 250 mg/d	61	585/148	RCT
				Doc 75mg/m2, q3w	60	583/150	
Garassino 2013 [21]	219	219	0	Erl 150 mg/d	66	90/19	RCT
				Doc 75mg/m2, q3w	67	80/30	
Kawaguchi 2014 [22]	301	199	56	Erl 150 mg/d	68	111/39	RCT
				Doc 60mg/m2, q3w	67	114/37	

EGFR^+^: presence of epidermal growth factor receptor mutation; EGFR^−^: absence of epidermal growth factor receptor mutation; Ate: atezolizumab; Pem: pembrolizumab; Doc: docetaxel; Gef: gefitinib; Erl: erlotinib; -: not available; N: the total number of patients; n: the number of patients with known EGFR status; RCT: randomised controlled trials.

### Comparison of anti-PD-1/PD-L1 therapy vs. docetaxel

**Figure 2:**
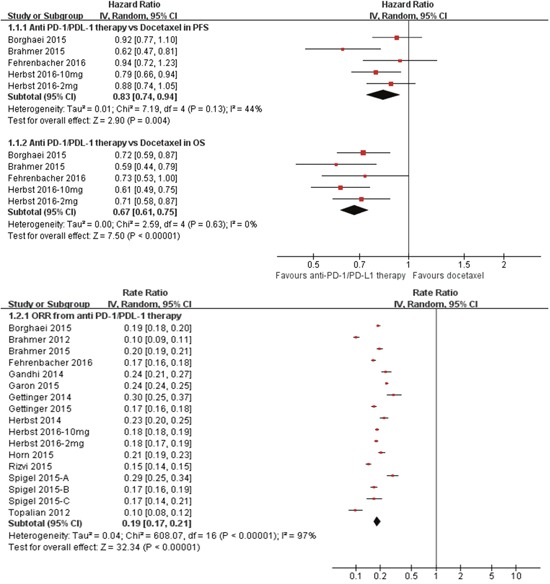
Meta-analysis of the treatment effects of anti-PD-1/PD-L1 therapy in patients with advanced non-small cell lung cancer (1.1) anti-PD-1/PD-L1 therapy vs. Docetaxel in progression free survival (PFS) and overall survival (OS);(1.2) overall response rate (ORR) from anti-PD-1/PD-L1 therapy. CI: 95 % confidence interval; Random: random-effects model.

**Figure 3:**
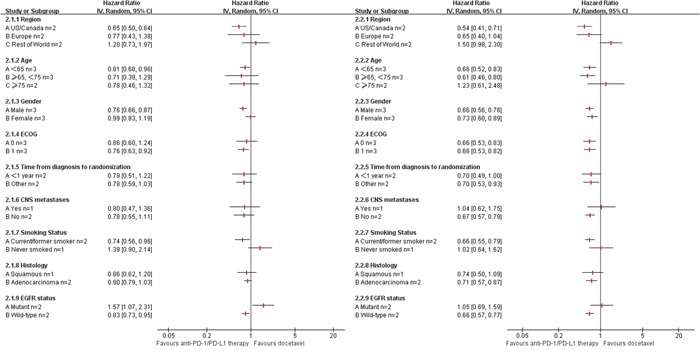
Subgroup Meta-analysis of progression free survival (PFS) and overall survival (OS) (2.1) anti-PD-1/PD-L1 therapy vs. Docetaxel in PFS; (2.2) anti-PD-1/PD-L1 therapy vs. Docetaxel in OS. ECOG: Eastern Cooperative Oncology Group performance status; CI: 95 % confidence interval; Random: random-effects model.

In the total population, the pooled analysis indicated that anti-PD-1/PD-L1 therapy consistently reduced the risk of death by 33% over docetaxel (hazard ratio [HR] 0.67, P<0.001), and prolonged the PFS by 17%(HR 0.83, P<0.001) (Figure 2). The values for heterogeneity tests across these trials were *I*^2^=0%, P=1.00 for OS analysis; *I*^2^=44%, P=0.13 for PFS analysis. The HRs in this analysis of OS favored anti-PD-1/PD-L1 therapy across most prespecified subpopulation; the exceptions were the subpopulation who lived in the rest-of-the-world geographic region, those with age more than 75 years, those with central nervous system metastases, those who had never smoked, and those with EGFR mutation(Figure 3). And, similar results was shown in PFS analysis(Figure 3). And, anti-PD-1/PD-L1 therapy resulted in an impressive ORR of 19%(Figure 2). No significant publication bias was found in the ORR analysis (P= 0.582). The value for heterogeneity test across these trials was *I*^2^=97%, P<0.001 for ORR analysis.

**Figure 4:**
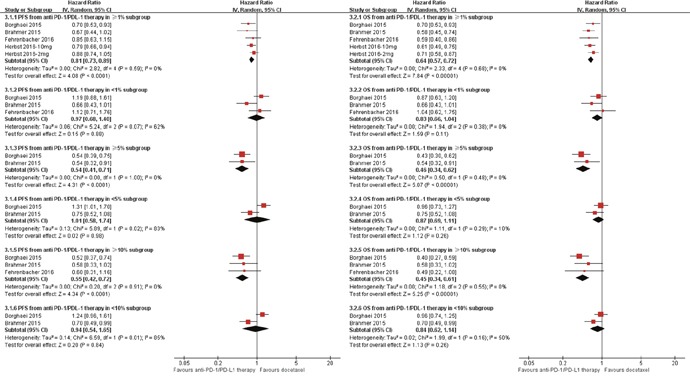
Meta-analysis of progression free survival (PFS) and overall survival (OS) by PD-L1 Expression Level (3.1) anti-PD-1/PD-L1 therapy vs. Docetaxel in PFS; (3.2) anti-PD-1/PD-L1 therapy vs. Docetaxel in OS. CI: 95 % confidence interval; Random: random-effects model.

Subgroup analyses by the tumor PD-L1 expression level indicated that anti-PD-1/PD-L1 therapy could significantly prolong both PFS and OS in patients of high PD-L1 expressions, but not in those with low expressions, regardless of PD-L1 expression level of 1%, 5%, and 10% (Figure 4). Furthermore, this analysis declared statistically significant difference between PD-L1 expression level of≥5% and <5% (P=0.0008), between PD-L1 expression level of≥10% and <10%(P=0.005) in OS analysis. And, PFS analysis indicated a marginal difference between PD-L1 expression level of≥5% and <5% (P=0.05).

**Figure 5:**
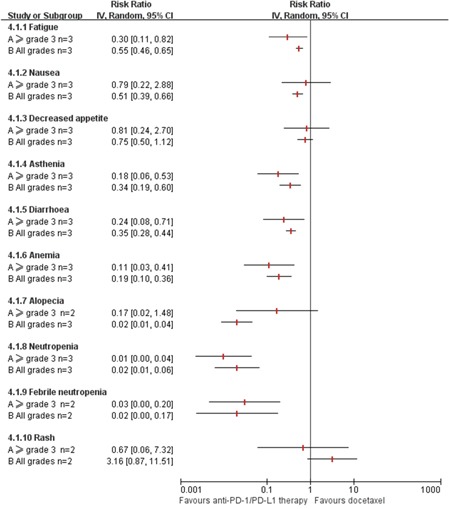
Meta-analysis of adverse events (AEs) of anti-PD-1/PD-L1 therapy vs. Docetaxel in previously treated patients with advanced non-small cell lung cancer n: the number of included trials for analysis; CI: 95% confidence interval; Random: random-effects model.

Generally, the rates of adverse events (AEs) of anti-PD-1/PD-L1 therapy seemed to be lower than that of docetaxel (Figure 5).

### Indirect comparison of anti-PD-1/PD-L1 therapy vs. EGFR-TKIs via common comparator of docetaxel

**Figure 6:**
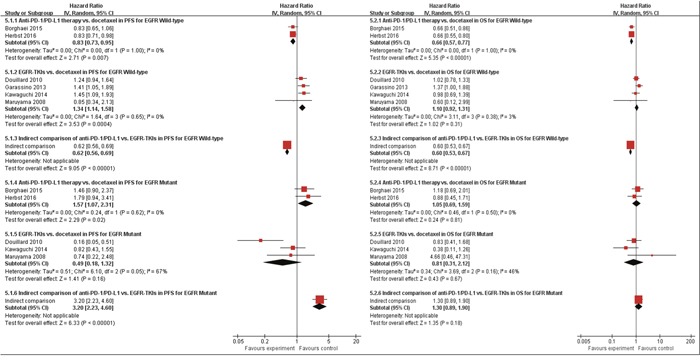
Indirect meta-analysis of treatment effects (anti-PD-1/PD-L1 therapy vs. EGFR-TKIs via common comparator) in progression free survival (PFS) (4.1.) and overall survival (OS) (4.2.) in previously heavily treated patients with and without EGFR mutation CI: 95 % confidence interval; Random: random-effects model.

When using docetaxel as common comparator, our indirect comparison indicated that anti-PD-1/PD-L1 could reduce the progression of risk by 38% (HR 0.62, P<0.001), and prolonged OS by 40% (HR 0.60, P<0.001) for those EGFR wild-type patients(Figure 6). This finding has special meaning, because a larger number of patients with advanced NSCLC are EGFR wild-type. Meanwhile, for those EGFR mutant patients, indirect comparison indicated that anti-PD-1/PD-L1 therapy was inferior to EGFR-TKIs therapy in terms of PFS (HR 3.20, P<0.001), but no survival difference between them (HR 1.30, P=0.18) (Figure 6).

## DISCUSSION

In this pooled analysis, an impressive ORR of 19% derived from anti-PD-1/PD-L1 therapy compared favorably to the ORRs of 7% to 9% from current second-line therapies for advanced NSCLC [24–26]. Furthermore, anti-PD-1/PD-L1 therapy clearly benefited patients over docetaxel concerning PFS and OS. These data contribute to the increasing evidence that supports PD-1 pathway inhibition in advanced NSCLC. Anti-PD-1/PD-L1 therapy related with fewer treatment-related adverse events than was docetaxel. Unlike chemotherapy, immune checkpoint inhibitors by blocking the PD-1 inhibitory receptor tried to restore antitumor immunity. So anti-PD-1/PD-L1 therapy added to chemotherapy might be a good option for such patients. Ongoing trials are assessing anti-PD-1/PD-L1 therapy as adjuvant therapy (PEARLS, ClinicalTrials.gov numberNCT02504372). These trials were enrolling patients using different biomarker cutpoints, and which cutpoint could best predict the activity of anti-PD-1/PD-L1 therapy still remains undefined. Furthermore, this analysis indicated statistically significant difference between PD-L1 expression level of≥5% and <5% (P=0.0008), between PD-L1 expression level of≥10% and <10% (P=0.005) in OS analysis. And, a marginal difference between PD-L1 expression level of≥5% and <5% (P=0.05) was shown in PFS analysis. Analysis by smoking history indicated favorable PFS and OS outcomes in former and current smokers, which could be explained by the expected higher mutational load in smoking-associated lung cancer. And there were separate anti-PD-1/PD-L1 therapy studies for squamous and non-squamous, this analysis indicated that anti-PD-1/PD-L1 therapy could provide similar benefit for squamous and non-squamous NSCLC.

Whether the benefit of anti-PD-1/PD-L1 therapy extends to patients with a tumour proportion score of less than 1% needs to be defined in future trials. KEYNOTE-024 trial [25] had indicated anti-PD-1/PD-L1 therapy used as first-line therapy could improve treatment outcome than chemotherapy. Another ongoing study is also assessing anti-PD-1/PD-L1 therapy as first-line therapy (KEYNOTE-042, ClinicalTrials.gov number NCT02220894). This study is enrolling patients using distinct biomarker cutpoints, and the final analyses could provide help to determine which cutpoint best predicts activity of anti-PD-1/PD-L1 therapy monotherapy in these earlier lines of therapy. For those patients for whom anti-PD-1/PD-L1 therapy monotherapy is not as effective as cytotoxic chemotherapy, in combination with chemotherapy [26] or other immunotherapies [27] might be needed. Additional studies will also be needed to define the optimal duration of anti-PD-1/PD-L1 therapy.

Responses with anti-PD-1/PD-L1 therapy were seen in patients with EGFR- and KRAS-wildtype and EGFR- and KRAS-mutant NSCLC; however, low numbers of enrolled patients in these trials precluded relationship of mutation status with clinical outcomes after anti-PD-1/PD-L1 therapy. Futhermore, the introduction of anti-PD-1/PD-L1 therapy and EGFR-TKIs for heavily pretreated patients with NSCLC had also created a dilemma regarding whether anti-PD-1/PD-L1 agent was better than EGFR-TKIs, or vice versa. Using the common comparator of docetaxel, our indirect comparison indicated that PFS and OS with anti-PD-1/PD-L1 were superior to that with EGFR-TKIs. Meanwhile, for those EGFR mutant patients, anti-PD-1/PD-L1 therapy was inferior to EGFR-TKIs therapy in terms of PFS.

However, these findings should be viewed with caution: First caveat is that of relatively insufficient evidence because of the limitation of indirect comparison [23]. So, a direct head-to-head trial comparing anti-PD-1/PD-L1 therapy versus EGFR-TKIs is clearly warranted in future. Secondly, we used abstracted data, whereas an individual patient data-based analysis would provide a more precise estimate of the activity and safety of anti-PD-1/PD-L1 therapy versus EGFR-TKIs. Thirdly, these studies were relatively heterogeneous with respect to patient population, disease status, and study design. For our primary outcomes analysis, the heterogeneity among selected studies were were low and non-significant for PFS (P=0.13, *I*^2^=44%) and OS (P=0.63, *I*^2^=0%) analysis, high and significant for ORR (P<0.001, *I*^2^=97%) analysis (Figure 2). Given this high and significant difference among these included trials for ORR analysis, the pooled ORR could be questioned.

In summary, anti-PD-1/PD-L1 therapy could produce clinical benefit over docetaxel for patients with previously treated NSCLC. For these EGFR wild-type patients, anti-PD-1/PD-L1 therapy seemed to prolong PFS and OS when compared with EGFR-TKIs. Meanwhile, among those EGFR mutant patients, anti-PD-1/PD-L1 therapy was inferior to EGFR-TKIs therapy in terms of PFS.

## MATERIALS AND METHODS

### Literature search strategy

The Cochrane Controlled Trial Register, Embase, Medline, and the Science Citation Index were searched using the medical subject headings “lung cancer”, Atezolizumab”, “Pembrolizumab”, “Nivolumab”, “Gefitinib” and “Erlotinib”. Reference lists of selected reports were also hand-searched. This pooled analysis was approved by the institutional review boards of Weifang People's Hospital, in accordance with the Helsinki Declaration.

### Selection of studies

Trials were included for this analysis if they met the following criteria: (1) They dealt only with previously treated advanced NSCLC patients. (2) They enrolled patients treated with anti-PD-1/PD-L1 therapy or EGFR-TKIs.(3) Acceptable comparator was docetaxel. (4) They could provide data about adverse events rate, response rate, overall survival (OS) and (or) progression free survival (PFS). (5) These studies are prospective. Multiple reports about a single trial were considered as one. All potential trials were reviewed by two investigators separately (Y.X.Z and Z.X.S.).

### Quality assessment

Two reviewers (Y.X.Z and Z.X.S.) independently assessed the quality of selected RCTs using the following criteria: (1) generation of allocation sequence, (2) description of dropouts, (3) masking of randomization, intervention, outcome assessment, (4) intention-to-treat analyses. Each criterion was rated as yes, no or unclear.

### Outcome measures

The primary objective was to define the Hazard Ratios (HRs) of PFS and OS for anti-PD-1/PD-L1 therapy versus EGFR-TKIs, and calculate ORR from anti-PD-1/PD-L1 therapy. The secondary objectives were to evaluate the Risk Ratio (RRs) of adverse events for anti-PD-1/PD-L1 therapy versus docetaxel.

### Statistical analysis

The indirect meta-analysis preserves the randomization within a RCT meanwhile, combines all available comparisons between treatments [23, 24]. These comparisons included both the direct within trial comparisons between two treatment strategies and the indirect comparisons constructed from trials that have one common comparator. When more than one RCT was available for comparison (e.g., anti-PD-1/PD-L1 therapy vs. Docetaxel), we first calculated the pooled estimates using standard meta-analytic techniques for that comparison. Using similar method, we obtained a pooled estimate from RCTs that compared other interventions (e.g., EGFR-TKIs vs. Docetaxel). Because both comparisons used docetacel as control, the summary estimates obtained from the respective meta-analysis (anti-PD-1/PD-L1 therapy vs. Docetaxel and EGFR-TKIs vs. Docetaxel) can be used to provide estimates of the HR for the indirect comparison of anti-PD-1/PD-L1 therapy vs. EGFR-TKIs. The adjusted indirect comparisons were performed using the method described by Bucher et al [24]. According to this, an indirect comparison of interventions anti-PD-1/PD-L1 therapy vs. EGFR-TKIs can be obtained by adjusting the results of their direct comparisons with a common intervention of docetacel. If we assume that anti-PD-1/PD-L1 therapy_MA_ is the estimate of direct comparison between intervention anti-PD-1/PD-L1 therapy vs. docetacel and EGFR-TKIs_MA_ is the direct comparison of intervention EGFR-TKIs vs. docetacel, then the estimate of the adjusted indirect comparison of intervention EGFR-TKIs vs. docetacel (such as log HR) is estimated by anti-PD-1/PD-L1 therapy_MA_-EGFR-TKIs_MA_ [23, 24]. Because the estimates are obtained from different studies, the results are statistically independent and its variance can be obtained by Var (log (anti-PD-1/PD-L1 therapy_MA_) + Var (log (EGFR-TKIs_MA_) [23, 24].

All these analyses were undertaken using a random-effects model which could provided a more conservative result. The heterogeneity among these trials was evaluated using Cochrane χ^2^ test and quantified with the *I*^2^ statistic. Statistical heterogeneity was considered significant when the test produced a P-value <0.1. The *I*^2^ statistic was calculated as a measure of the degree of heterogeneity among selected studies, where *I*^2^ values of 25%, 50% and 75% were considered low, moderate and high degrees of heterogeneity respectively. We also undertook subgroup analyses to sought the source of heterogeneity. Publication bias was evaluated with Egger's test. All meta-analyses were undertaken with Review Manager (version 5.3; The Cochrane Collaboration, Oxford, England) and Stata ver. 12.0 software (College Station, TX). Statistical significance was defined as a P value of less than 0.05 except for heterogeneity test.

## Abbreviations

NSCLC: non-small-cell lung cancer
PD-1: programmed death 1
PD-L1: programmed death-ligand 1
PFS: progression free survival
OS: overall survival
EGFR-TKIs: epidermal growth factor receptor tyrosine kinase inhibitors
ORR: overall response rate
AEs: adverse events
